# Epididymitis

**Published:** 1892-07

**Authors:** 


EPIDIDYMITIS.—Dr. Brewer, following the younger Langlebert, has employed in the Vanderbilt clinic what he calls the dry poultice in some twenty-five or thirty cases of epididymitis of both tubercular and gonorrhœal origin. The dressing consists of a moderately thick layer of cotton wool, applied over the testicle, covered by a layer of thin rubber protective, so fashioned as to completely cover the diseased organ, and extend up onto the sound tissue, partially enclosing the sound side. This is secured by a snugly-applied bandage, and the whole held in place by a suspensory. Its results, as regards the absorption of the induration, are rapid and marked, a very considerable diminution being seen at each dressing, and the author adds that no other method of treatment has in his hands proved so satisfactory.—*Jour. Cutan. and Ven. Diseases.*

